# Identification of extracellular vesicles and characterization of miRNA expression profiles in human blastocoel fluid

**DOI:** 10.1038/s41598-018-36452-7

**Published:** 2019-01-14

**Authors:** R. Battaglia, S. Palini, M. E. Vento, A. La Ferlita, M. J. Lo Faro, E. Caroppo, P. Borzì, L. Falzone, D. Barbagallo, M. Ragusa, M. Scalia, G. D’Amato, P. Scollo, P. Musumeci, M. Purrello, E. Gravotta, C. Di Pietro

**Affiliations:** 1 Department of Biomedical and Biotechnological Sciences, University of Catania, Catania, Italy; 2 Reproductive and IVF Unit- PTA “F Jaia”, Department of Maternal and Child Health, Conversano, Italy; 3 IVF Unit Cervesi Hospital Cattolica, Cattolica, RN Italy; 4 IVF Unit, Cannizzaro Hospital, Catania, Italy; 5 Dipartimento di Fisica e Astronomia, Università di Catania, Catania, Italy; 6 IPCF-CNR, viale F. Stagno d’Alcontres 37, 98158 Messina, Italy; 7 Oasi Research Institute - IRCCS, Troina, Italy; 8 Merck Serono s.p.a. Medical Affairs Department, Fertility, Endocrinology and General Medicine, Roma, Italy

## Abstract

In this study, for the first time, we demonstrated the presence of microRNAs and extracellular vesicles in human blastocoel fluid. The bioinformatic and comparative analyses identified the biological function of blastocoel fluid microRNAs and suggested a potential role inside the human blastocyst. We found 89 microRNAs, expressed at different levels, able to regulate critical signaling pathways controlling embryo development, such as pluripotency, cell reprogramming, epigenetic modifications, intercellular communication, cell adhesion and cell fate. Blastocoel fluid microRNAs reflect the miRNome of embryonic cells and their presence, associated with the discovery of extracellular vesicles, inside blastocoel fluid, strongly suggests their important role in mediating cell communication among blastocyst cells. Their characterization is important to better understand the earliest stages of embryogenesis and the complex circuits regulating pluripotency. Moreover, blastocoel fluid microRNA profiles could be influenced by blastocyst quality, therefore, microRNAs might be used to assess embryo potential in IVF cycles.

## Introduction

During cavitation, at day 4 of human preimplantation development, embryo cells begin to differentiate into the Inner Cell Mass (ICM) and Trophectoderm (TE) lineages and secrete fluid into the morula to create a fluid-filled cavity, the blastocoel. As the embryo further divides, the blastocoel expands and the ICM becomes positioned on one side of the trophoblast cells forming the mammalian blastula, called blastocyst, ready for implantation^1^. In assisted reproductive cycles, over the last few years, extended embryo culture up to the blastocyst stage is widely practiced to improve pregnancy rates and reduce the probability of multiple pregnancies^2^. Of course, the assessment of blastocyst quality represents the basic step to achieve a successful pregnancy. In spite of numerous papers suggesting time-lapse microscopy, as well as biochemical and molecular analyses to detect the most suitable embryo, to date, in clinical applications, morphological evaluation is the most accepted method to assess embryo quality^3^. For this purpose, several morphological scoring systems, mainly based on the expansion of the blastocoel cavity, as well as on the appearance of the ICM and TE cells, have been proposed^4–6^. In addition to these conventional methods of embryo evaluation, preimplantation genetic screening (PGS) in IVF cycles has been introduced as a valuable tool aimed at choosing euploid embryos to improve pregnancy rates^7^. Moreover, to prevent the transmission of single gene disorders such as cystic fibrosis and β-thalassemia, different methods of preimplantation genetic diagnosis (PGD) have been developed^8^. Even though the premise behind PGS and PGD is widely accepted, the safety of the biopsy stage, involving the invasive removal of cells from the TE, is still considered a critical aspect^9^. In light of this evidence, the identification of specific markers for the choice of high-quality embryos, in a minimally invasive manner, represents one of the most intriguing challenges for contemporary medicine.

In 2013, for the first time, genomic DNA was identified inside Blastocoel Fluid (BF) and the authors proposed that BF could represent a good option for PGD avoiding the potential risk associated with embryo biopsy^10^. Further studies, by Whole Genome Amplification (WGA), showed a high level of concordance (97%) with TE biopsy demonstrating the potential use of the BF DNA also for aneuploidy detection and, in general, for PGS^11,12^. In spite of the unequivocal presence of DNA fragments in BF, their origin is unknown. Genomic and Mitochondrial DNA have been detected in embryo culture medium and, as well as BF DNA, these DNA fragments could represent potential targets for PGD or PGS. However, compared with BF, there is a higher risk of extra-embryonic DNA contamination within the spent culture medium^13^.

In addition to the potential use of DNA fragments to investigate embryo health, it has recently been proposed that microRNAs (miRNAs) could represent molecular markers of blastocyst quality. In fact, miRNAs have been found in the spent culture medium, their expression profiles reflected embryo aneuploidies and these could also be used to estimate embryo implantation potential^14,15^. MiRNAs are critical regulators of early embryonic development, they are able to maintain the embryonic stem cell self-renewal and, at the same time, induce cellular differentiation^16^. It has been demonstrated that early embryos synthesize miRNAs to participate in the regulatory circuitry controlling stemness and differentiation and are also able to secrete miRNAs outside the blastocyst, possibly to mediate the dialog between embryo and endometrium^14,15^. In general, secreted miRNAs can be free or enclosed inside extracellular vesicles (EVs) as microvesicles or exosomes. EVs play an important role in intercellular communication carrying and transferring, not only miRNAs, but also different molecules as proteins, lipids, mRNAs, non-coding RNAs and DNA to recipient cells^17^. Two recent papers have shown that extracellular vesicles secreted by blastocysts in culture medium are taken up by endometrial epithelial cells and although these papers did not characterize the molecule cargo, they certainly demonstrated that embryonic cells use microvesicles and exosomes to communicate with maternal tissues^18,19^.

If embryo cells are able to produce and secrete miRNAs in culture medium *in vitro* and in the uterus *in vivo*, it would seem logical to assume that extracellular vesicles carrying miRNAs, DNA and other molecules, should be present in BF and that the analysis of their cargo could reflect embryo quality. In this study, for the first time, we demonstrated the presence of miRNAs and exosomes in human BF. By bioinformatic and comparative analyses, we investigated the biological function of the identified miRNAs and suggested their potential role inside blastocyst. The discovery of miRNAs and exosomes in BF represents the further confirmation of the importance of cell communication mechanisms mediated by extracellular vesicles through their cargo. BF miRNA characterization is important to understand the biology of human embryonic stem cells. Moreover, BF miRNA profiles could be influenced by blastocyst quality; therefore, these miRNAs might be used to assess embryo potential in IVF cycles.

## Results

### MicroRNA identification and characterization

**Figure 1:**
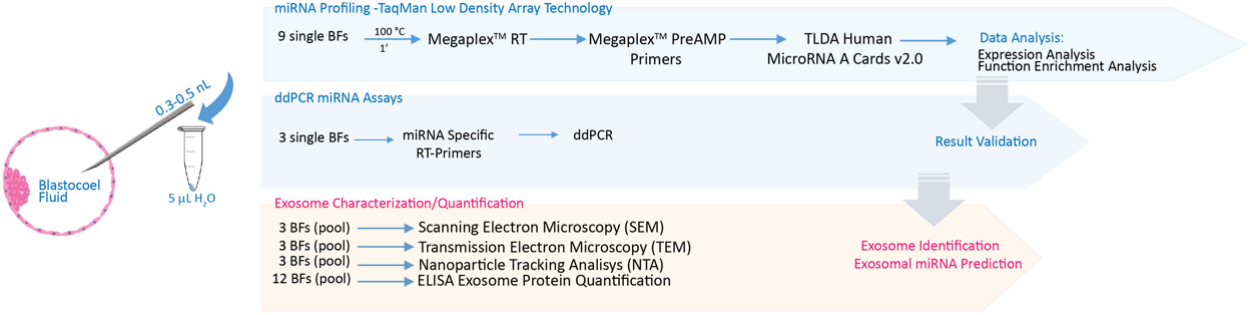
Schematic overview of experimental workflow for miRNA profiling and exosome characterization in human Blastocoel Fluid.

**Figure 2:**
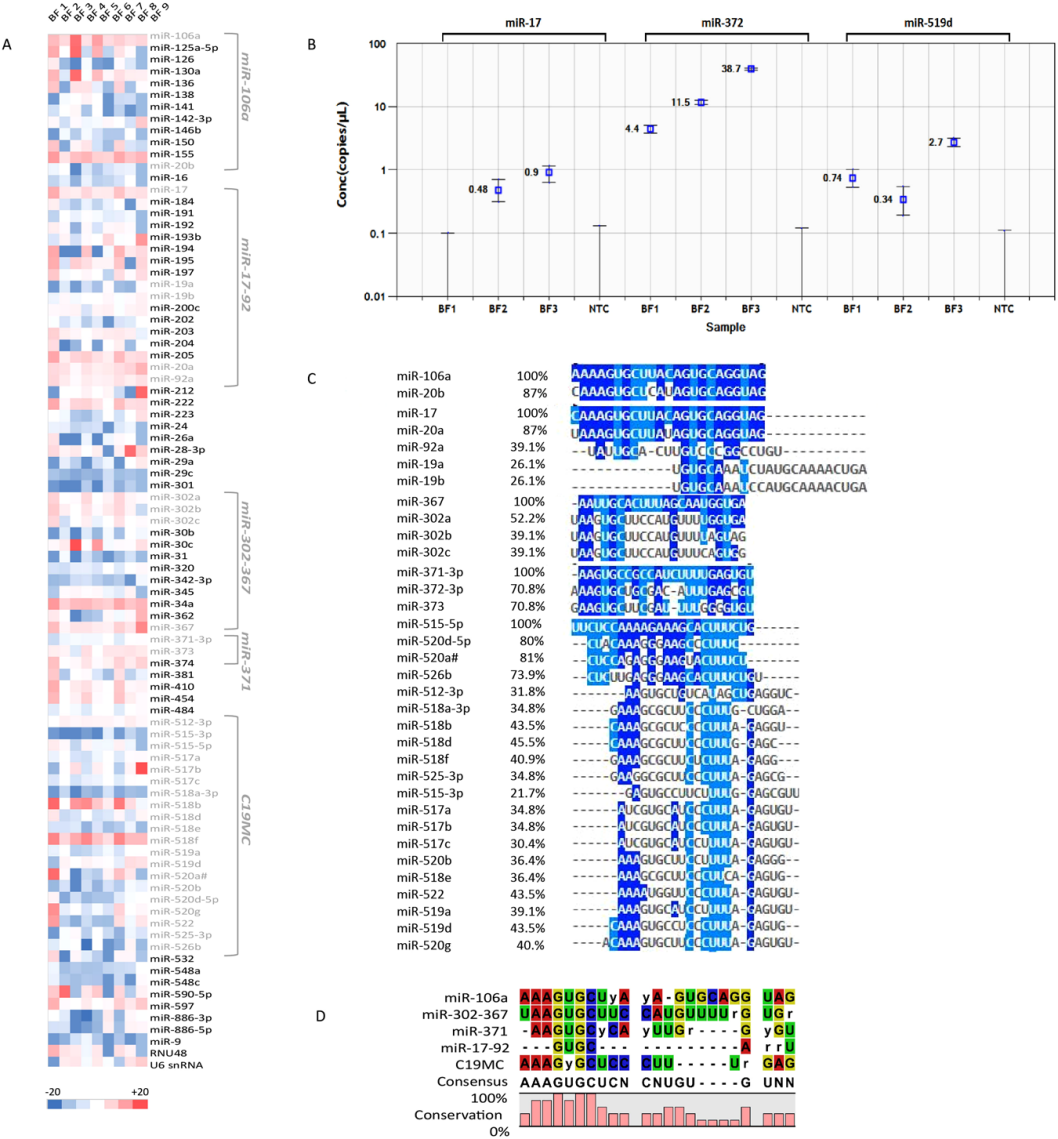
miRNA expression in human Blastocoel Fluid (BF). (**A**) Heat map representation of normalized expression data (−ΔCT values) for 89 miRNAs from nine BF samples. The red and blue colors represent miRNA expression levels. (**B**) Quantification of BF miRNAs using droplet digital PCR (ddPCR). Measurements for miR-17, miR-372 and miR-519d are shown as miRNA copies/μl ddPCR mix. All NTC controls do not show positive droplets. (**C**) Alignment of human BF miRNAs. Blue shading indicates nucleotides of miRNA sequences with identical positions in the alignment. Light blue shading indicates nucleotides conserved in the major groups of BF miRNAs. (**D**) Consensus clustering and frequency of nucleotide conservation for groups of BF-miRNAs. Most of the mature miRNAs conserve the seed sequence AAGUGC.

We identified 89 miRNAs in BF by the analysis of nine single human blastocysts. The methods used for the identification have been summarized in Fig. 1. MiRNA expression levels, normalized for miR-372 in the nine BF are expressed as negative ΔCt values (Fig. 2A). For some of them, the expression level seems to be variable among the different samples (−20 < −ΔCT < 20), on the contrary, many of them present a more constant expression (Fig. 2A, Table S1). Additionally, miR-17, miR-519d and miR-372 absolute quantification, by droplet digital PCR (ddPCR), demonstrated that BF miRNAs were effectively detectable in the 3 analyzed samples. In particular, miR-372, used as housekeeping gene in Real- Time PCR experiments, showed higher concentration levels (ranging from 4.4 to 38.7 copies/μl among the analyzed samples) compared to the other miRNAs. For miR-17 and miR-519d 0.0, 0.48, 0.9 and 0.74, 0.34, 2.7 copies/μl were detected, respectively (Fig. 2B, Table S2). No Template Controls (NTC) did not show positive droplets (Fig. 2B). Supplementary Table S2 reports all the data generated by the ddPCR analysis.

In agreement with data in the literature, all embryonic miRNA clusters were well represented: we found miR-302a, miR-302b, miR-302c and miR-367-3p members of embryo miR-302/367 cluster, miR-371a, miR-372 and miR-373 members of eutheria-specific miR-290/miR-371 cluster, miR-17, miR-19a, miR-19b, miR20a and miR-92a members of miR17-92a-1 cluster, known as oncomiR cluster and miR-20b and miR-106a located in miR-106a-363 cluster^20,21^. We also found 20 miRNA members of the large primate-specific microRNA gene cluster (C19MC)^22^ (Fig. 2A). Alignment of the sequences of the mature miRNAs belonging to these five chromosome clusters highlighted the conservation of the embryo miRNA motif in most of the identified miRNAs^20^ (Fig. 2C,D).

**Figure 3:**
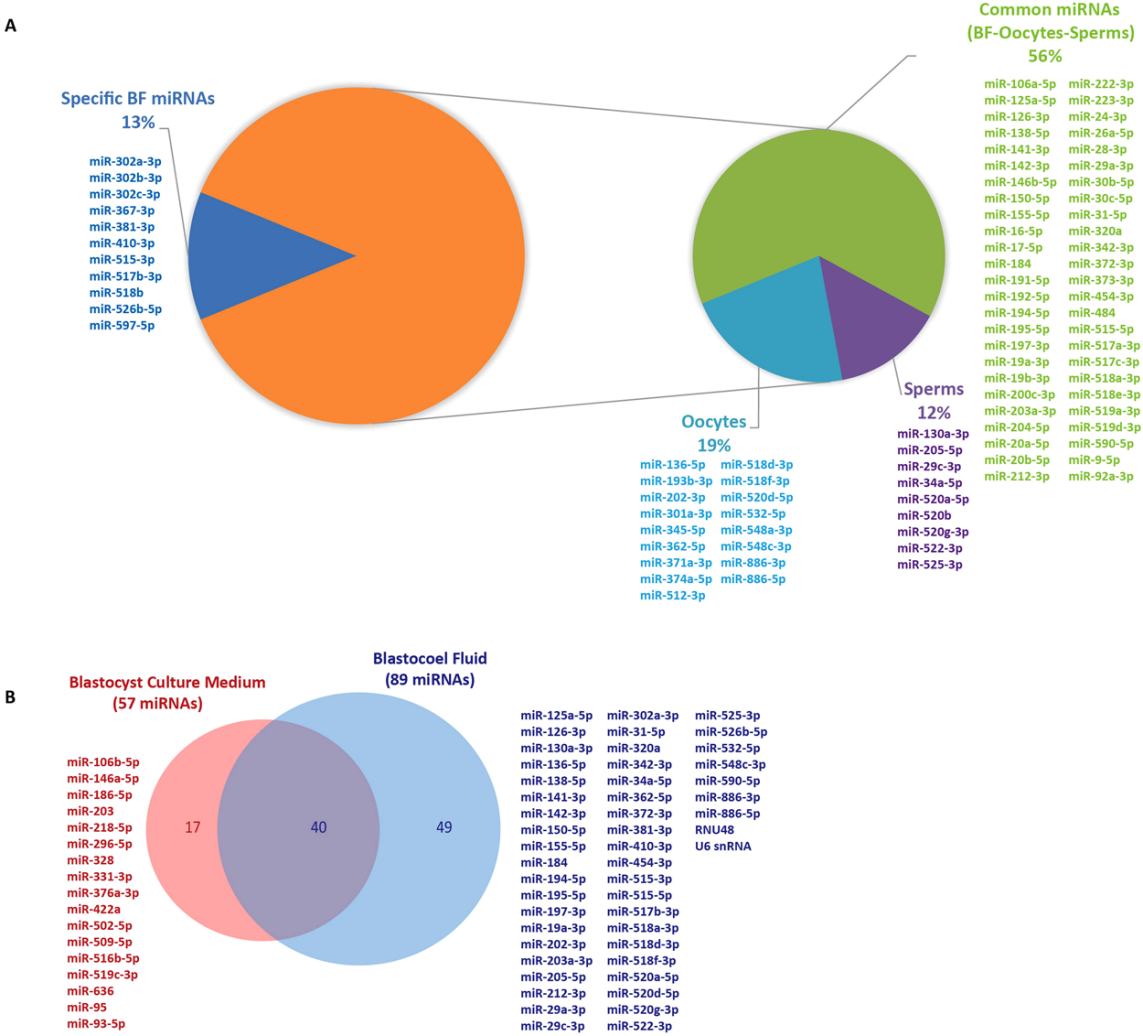
Comparison of BF miRNA expression profile in human oocyte, sperm and blastocyst culture medium. (**A**) Pie charts representing miRNAs from BF identified in human oocytes and sperms. Most of the detected miRNAs (87%) were shared among human gametes. Their distribution is indicated in the small Pie chart. **(B**) Venn diagram comparing BF miRNA expression with culture medium miRNAs.

We overlapped BF miRNAs with miRNAs identified in human oocytes by our group and with sperm miRNAs retrieved from an online database, to highlight maternal and paternal contribution and identify the miRNAs specifically synthesized by preimplantation embryos^23,24^. As regards the identified miRNAs, 78 (87%) were identified in the gametes and the most of them in oocytes (Fig. 3A). Among the 11 specific BF miRNAs, we found miR-302a, miR-302b, miR-302c and miR-367-3p, members of embryo miR-302/367 cluster and five members of C19MC (Fig. 2A). We then compared BF miRNAs with miRNAs identified in embryo culture medium by Capalbo and coauthors (in this paper, the authors used the same technology as we did; TaqMan Low Density Array)^14^. We found 49 miRNAs specific of BF and not present in embryo culture medium and 17 miRNAs specific of culture medium and absent in BF (Fig. 3B). Finally, among the 89 identified miRNAs, we found that 80% of them were described in the ExoCarta database as exo-miRNAs (Table 1).

**Table 1:** BF miRNAs and Exosomes.

BF miRNAs identified in Exocarta				BF miRNAs absent in Exocarta	
1	miR-9-5p	37	miR-203a-3p	1	miR-202-3p
2	miR-16-5p	38	miR-204-5p	2	miR-367-3p
3	miR-17-5p	39	miR-205-5p	3	miR-372-3p
4	miR-19a-3p	40	miR-212-3p	4	miR-410-3p
5	miR-19b-3p	41	miR-222-3p	5	miR-515-3p
6	miR-20a-5p	42	miR-223-3p	6	miR-515-5p
7	miR-20b-5p	43	miR-301a-3p	7	miR-518e-3p
8	miR-24-3p	44	miR-302a-3p	8	miR-518f-3p
9	miR-26a-5p	45	miR-302b-3p	9	miR-519a-3p
10	miR-28-3p	46	miR-302c-3p	10	miR-520a-5p
11	miR-29a-3p	47	miR-320a	11	miR-520d-5p
12	miR-29c-3p	48	miR-342-3p	12	miR-522-3p
13	miR-30b-5p	49	miR-345-5p	13	miR-525-3p
14	miR-30c-5p	50	miR-362-5p	14	miR-548a-3p
15	miR-31-5p	51	miR-371a-3p	15	miR-548c-3p
16	miR-34a-5p	52	miR-373-3p	16	miR-597-5p
16	miR-92a-3p	53	miR-374a-5p	17	miR-886-5p
18	miR-106a-5p	54	miR-381-3p	18	RNU48
19	miR-125a-5p	55	miR-454-3p		
20	miR-126-3p	56	miR-484		
21	miR-130a-3p	57	miR-512-3p		
22	miR-136-5p	58	miR-517a-3p		
23	miR-138-5p	59	miR-517b-3p		
24	miR-141-3p	60	miR-517c-3p		
25	miR-142-3p	61	miR-518a-3p		
26	miR-146b-5p	62	miR-518b		
27	miR-150-5p	63	miR-518d-3p		
28	miR-155-5p	64	miR-519d-3p		
29	miR-184	65	miR-520b		
30	miR-191-5p	66	miR-520g-3p		
31	miR-192-5p	67	miR-526b-5p		
32	miR-193b-3p	68	miR-532-5p		
33	miR-194-5p	69	miR-590-5p		
34	miR-195-5p	70	miR-886-3p		
35	miR-197-3p	71	snRNA-U6		
36	miR-200c-3p				

### Gene Ontology and Pathway Analysis

**Figure 4:**
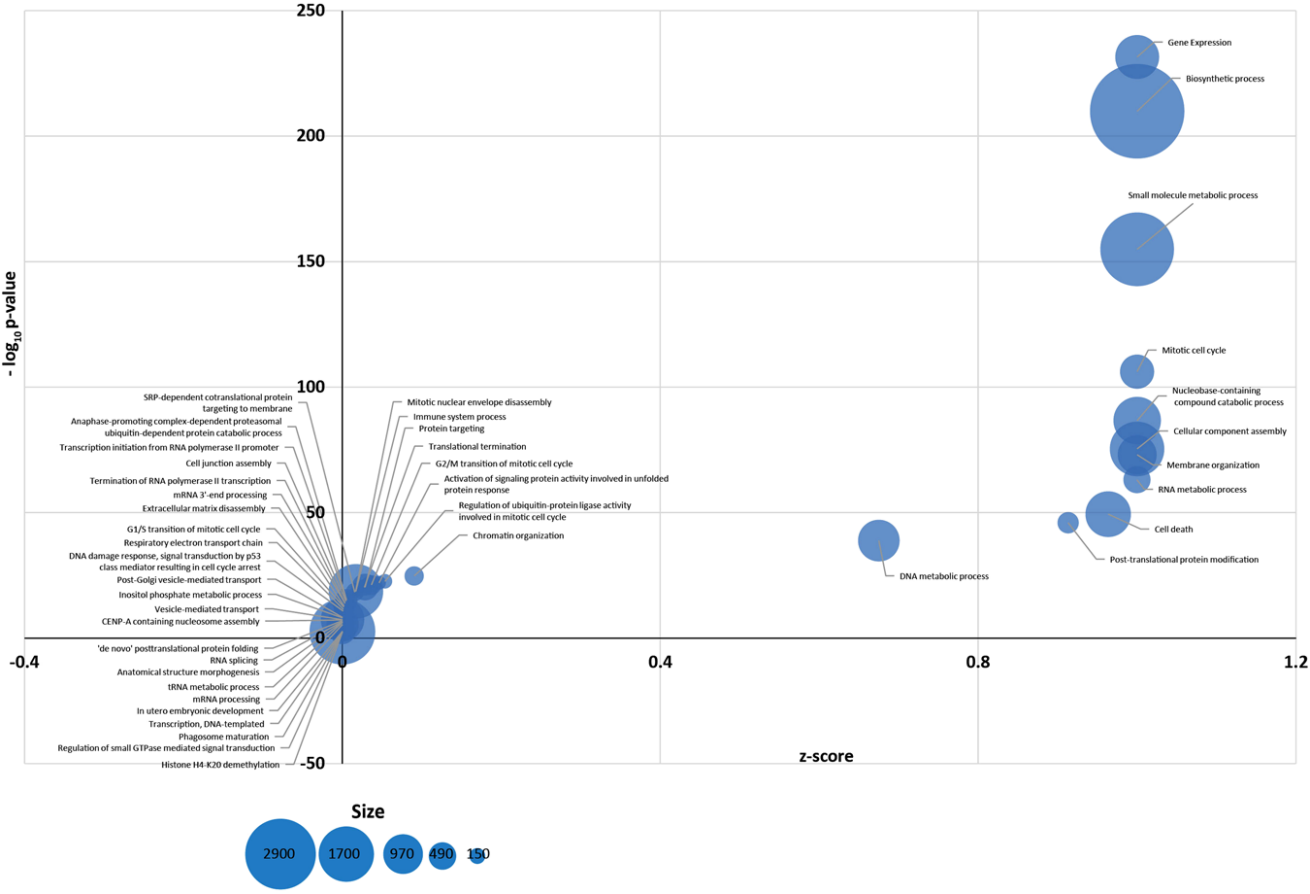
Significant GOs, biological processes, for miRNAs identified in human BFs. Bubble chart representing the most enriched functional categories for miRNA targets in BF. The y-axis represents the -log_10_ (P-value) and the x-axis the corresponding z-scores. The radius of the bubble is proportional to the size of the mRNA functional category (in terms of number of genes).

**Figure 5:**
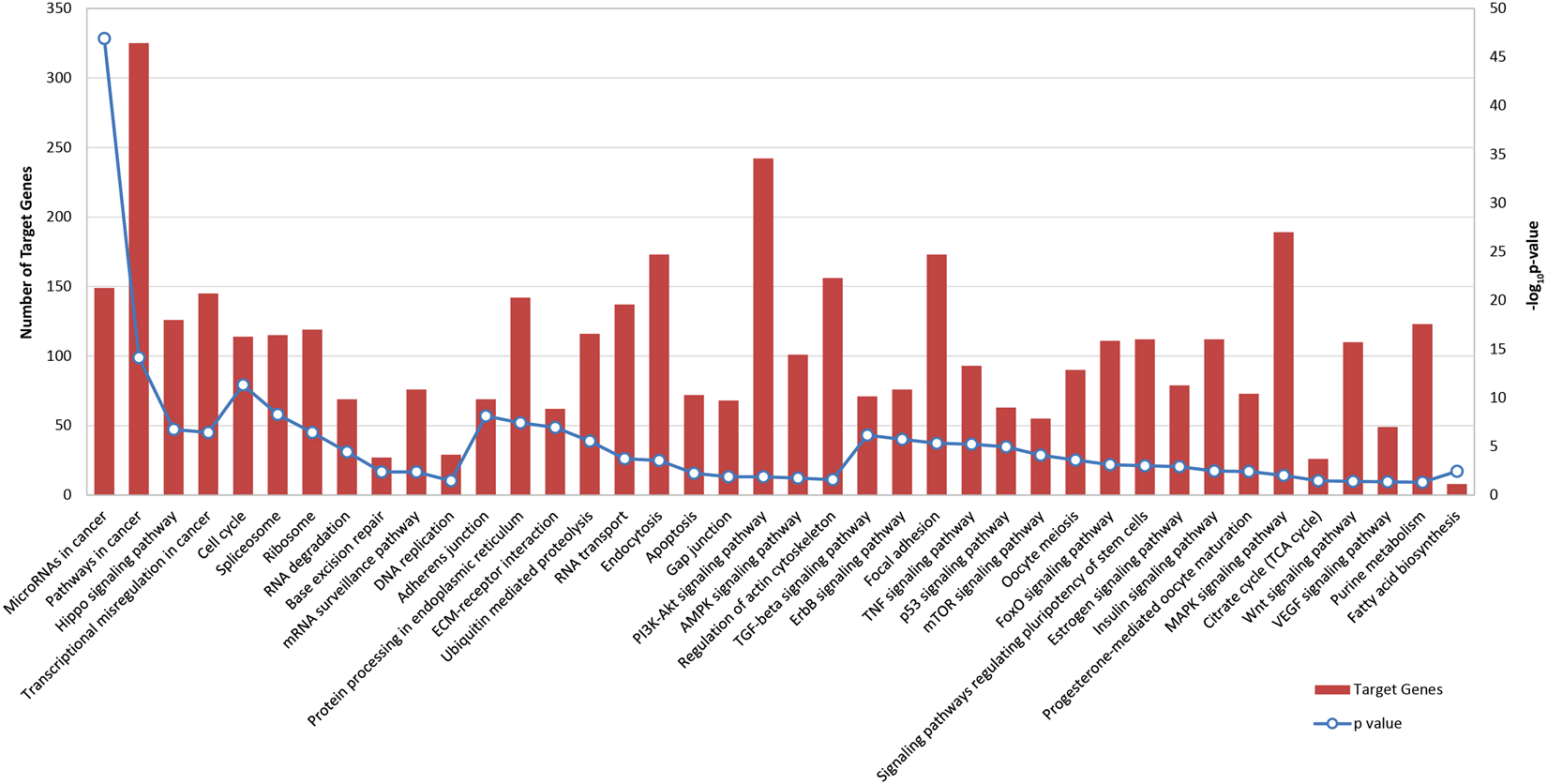
Signaling Pathway enrichment analysis for BF miRNAs with KEGG. Histograms representing pathways enriched in BF miRNA target genes. The probability values are reported as −log10 (P-value).

To explore the regulatory function of BF miRNAs, Gene Ontology (GO) analysis on validated miRNA targets was performed to functionally categorize miRNA target genes in a range of biological processes. The most significant biological processes include gene expression, biosynthetic process, small molecule metabolic process, mitotic cell cycle, cellular component assembly, cell death and post-translational protein modification (Fig. 4). Then KEGG Pathway analysis indicated that *Signaling pathways regulating pluripotency of stem cells*, *Hippo signaling pathway, Cell Cycle*, *Apoptosis*, *Gap junction* and *ECM-receptor interaction* were the most significant for the mRNA targets of BF miRNAs (Fig. 5).

### Extracellular Vesicle Characterization

**Figure 6:**
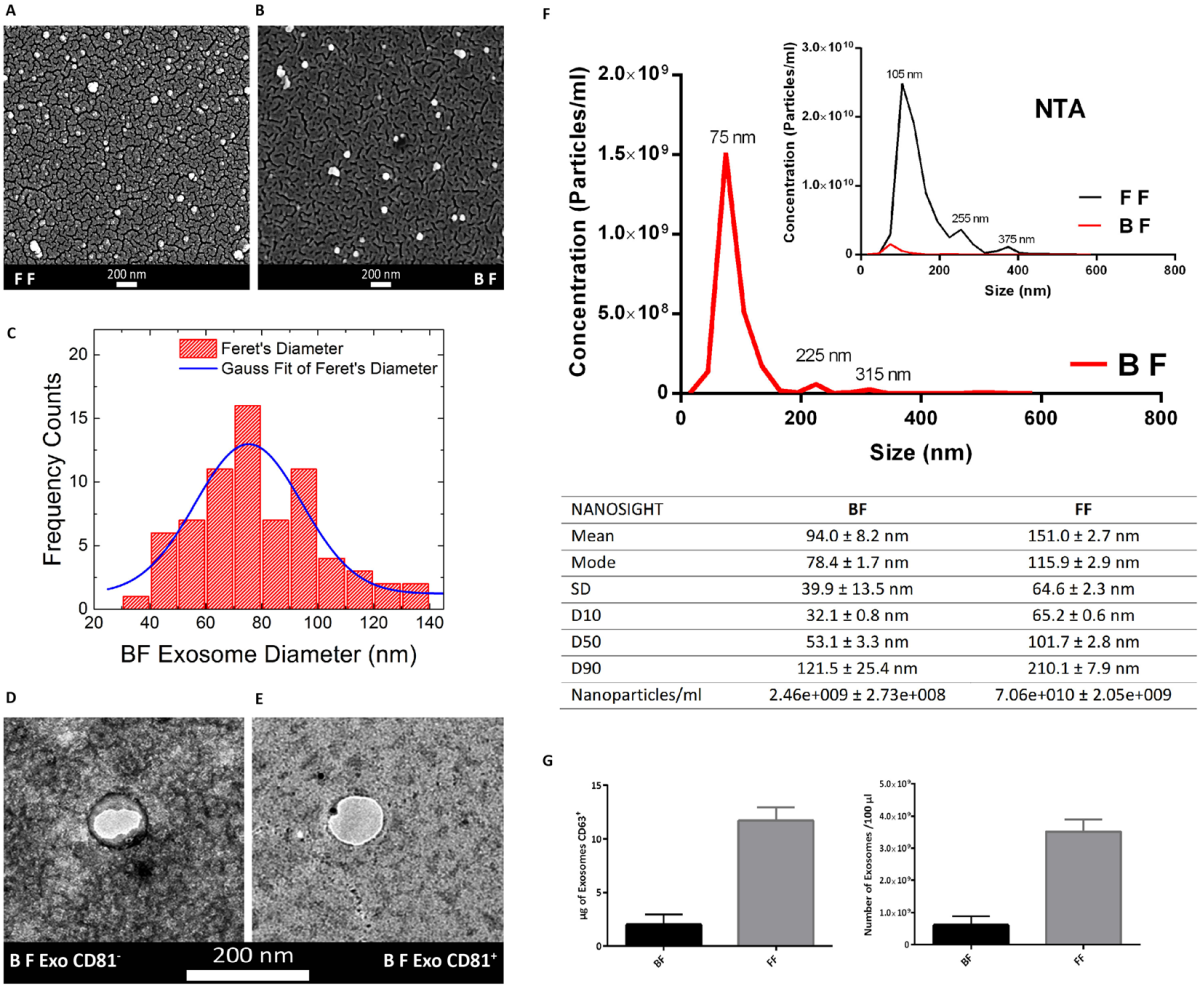
Morphological and Molecular Characterization of Exosomes from human Blastocoel Fluid. (**A**,**B**) Scanning Electron Micrographs of extracellular vesicles isolated from Follicular Fluid (FF) and Blastocoel Fluid (BF) respectively. (**C**) Diameter distribution of exosomes from BFs. Gauss fit of the Feret’s diameter histogram measured on SEM microscopies show an average BF diameter of 75 ± 3 nm and a full width at half maximum (FWHM) of 38 ± 8 nm. (**D**) Transmission Electron Microscopy images of exosomes from BFs. (**E**) Transmission Electron Microscopy images of exosomes from BFs marked with CD81. (**F**) Nanoparticle Tracking Analysis (NTA) of BF extracellular vesicles. Extracellular vesicles from Follicular Fluid were used as reference control (inset). Diameters and concentration of vesicles are indicated in the table. (**G**) ELISA assay with the tetraspanin CD63 antibody of BF exosomes. Amount (µg) of CD63 protein and EV concentration (number of particles/100 μl) evaluated in BFs. Follicular Fluid (FF) samples were used as reference control. Results are expressed as mean ± SEM.

We demonstrated the presence of exosomes in BF by morphological and molecular characterization. Morphological characterization has been performed by Scanning Electron Microscopy (SEM) and Nanoparticle Tracking Analysis (NTA). SEM observation revealed vesicles of spherical shape with an average diameter of 75 ± 3 nm and full width at half maximum (FWHM) of 38 ± 8 nm, compatible with exosome size (Fig. 6B,C).

Nanoparticle Tracking Analysis (NTA) confirmed that most of the EVs present in BF had a diameter of 78.4 +/− 1.7 nm (mean 94.0 +/− 8.2 nm) (Fig. 6F). Exosomes purified from human follicular fluid (FF) have been used as positive control in SEM (Fig. 6A) and in NTA analysis (Fig. 6F). The comparison revealed similar size between the two samples and as expected, an unequivocally greater vesicle concentration in the FF (Fig. 6A,F).

Transmission Electron Microscopy (TEM) and Enzyme-linked immunosorbent assay (ELISA) were used to analyze the expression of exosomal markers CD81 and CD63 respectively. The results clearly confirmed the exosome presence inside BF (Fig. 6E,G).

Exosome concentration has been calculated by NTA and ELISA assay and shown in Fig. (6F,G). The two techniques are not comparable because NTA measure the number of vesicles with a specific diameter; on the contrary, ELISA detect specifically the exosomes CD63 positive. Moreover, because of the low amount of BF and the high dilution factor used for the analysis (3 BF samples 1:70 in PBS for NTA and 12 BF samples 1:200 in PBS), vesicle concentration may not be very reliable.

## Discussion

Preimplantation embryos secrete miRNAs and extracellular vesicles in culture medium and the latter can be taken up by maternal tissues^18,19^. These findings suggested that miRNAs secreted outside the blastocyst could mediate the dialogue between embryo and endometrium *in vivo*^25^.

In this study, we showed the presence of miRNAs inside the blastocyst, in BF (Fig. 2), and demonstrated that BF miRNAs reflect, the miRNome of embryonic cells. Moreover, we identified EVs inside BF and by morphological and molecular characterization, we discovered the most of them are exosomes (Fig. 6).

The identified 89 miRNAs are able to regulate the typical embryo cell biological pathways. In fact, we found that most of the embryo miRNAs, already described, are well represented in BF^20,21^ (Fig. 2). We also detected 20 members of Chromosome 19 miRNA cluster (C19MC) that was previously described as expressed nearly exclusively in the placenta^22^. Interestingly, these miRNAs share the consensus sequences of embryo miRNAs, as shown in Fig. (2C,D). By alignment of the specific motifs present in the different clusters, we found a high degree of conservation, especially in the 5′ end, suggesting a functional role for these sequences (Fig. 2D)^26,27^. Among the identified miRNAs, we also found miRNAs implicated in epigenetic modification. We found miR29a-3p, miR-29c-3p, miR-193b-3p, miR-194-5p, and miR-200c-3p, targeting DNMT3A, miR-26a-5p targeting DNMT3B and miR-342-3p targeting HDAC9^28,29^. The negative regulation of DNMT3s and HDAC9 is essential to maintain an open chromatin structure and sustain the pluripotency of embryo cells. Moreover, it has been demonstrated that the miR-29 family is an important epigenetic regulator during human somatic cell reprogramming^30^.

In addition, miR-381, present in BF, seems to be involved in stemness, in fact, it is able to promote pluripotency via inhibition of multi-lineage differentiation and stimulation of self-renewal in embryonic stem cells (ESCs)^31^.

Gene Ontology analysis revealed that BF miRNAs regulate mRNAs involved in cellular processes related to stemness and cell communication (Fig. 4). On the other hand, pathway analysis predicted that BF miRNAs, in addition to *Signaling pathways regulating pluripotency*, *Cell Cycle* and *Apoptosis*, are involved in the regulation of pathways related to preimplantation embryo development such as *ECM-receptor interaction, Gap junction*, and the *Hippo signaling pathway* (Fig. 5). Extracellular matrix (ECM) and gap junction, as well as the Hippo signaling pathway, are involved in the first cell-fate decision leading to the formation of the TE, and the ICM. Compaction process represents the first event leading to the two different cell populations: the outer blastomeres will be selected to form the TE layer, whereas blastomeres that are situated inside will be selected to form the ICM^32^. The gap junctions, present in the plasma membrane and composed of different proteins of the connexin family, mediate the passage of signaling molecules and ions among embryonic cells. Cell to cell communication, mediated by these complexes, seems to be essential for the compaction^32,33^. On the other hand, cell fate specification, needed for the regulation of specific gene expression, is mediated by the Hippo pathway^34,35^. It has been demonstrated that, when the pathway is activated, two transcription factors (Yap and Taz) are phosphorylated and excluded from the nucleus. This prevents the transcription of target genes involved in TE commitment (i.e. CdX2). Therefore, the activation of Hippo signaling represses the TE fate and enhances ICM specification^34,35^.

The presence of microvesicles and exosomes inside blastocoels and the finding that most (80%) of identified miRNAs have been described as exomiRNAs, strongly suggest an alternative and active mechanism of cell to cell communication (Table 1, Fig. 6). It is known that EVs are able to transfer miRNAs and also different molecules (mRNAs, lncRNAs, DNA, lipids and proteins) among cells. Therefore, embryo miRNAs, present in BF, as the cargo of extracellular vesicles or as free-miRNAs, could act as paracrine or autocrine messengers sending different signals among blastocyst cells. BF miRNAs, involved in pluripotency, cell reprogramming, epigenetic modification, intercellular communication, cell adhesion and cell fate, could maintain the stemness of ICM, drive TE differentiation and regulate the synthesis, the secretion and the remodeling of ECM. The role of exosomes in developmental signaling has been demonstrated, even if the reported data concern more advanced developmental stages; a comprehensive review has been recently published^36^.

We found that most BF miRNAs had already been identified in gametes and the most of them in oocytes (Fig. 3A) and could represent an important starting point to investigate gamete competence. In spite of this, we cannot exclude that some of the miRNAs present in the gametes are also further synthesized by embryonic cells, but, certainly, the eleven BF specific miRNAs represent the first miRNAs synthesized by the embryo. Interestingly, among them, we found miR-302a, miR-302b, miR-302c and miR-367-3p, members of the embryo miR-302/367 cluster that represents the only embryo miRNA cluster absent in the gametes.

The overlapping of BF miRNAs with miRNAs described in embryo culture medium showed important different expression profiles between culture medium and BF (Fig. 3B). The differences suggest a different role of miRNAs inside the two compartments. We can suppose that miRNAs specifically secreted outside the blastocyst, in culture medium *in vitro* and in the uterus *in vivo*, could be involved in implantation, mediating the dialog between embryo and endometrium^18,19^, while specific BF miRNAs should regulate processes involved in embryo development.

## Conclusions

In this study, we demonstrated that BF miRNAs reflect the miRNome of preimplantation embryonic cells and suggest that BF miRNAs could represent molecular markers of blastocyst quality. DNA fragments have been discovered in BF and their use to explore embryo quality has been proposed^10^. To date, the origin of these DNA fragments is unknown. The presence of extracellular vesicles in BF could explain their derivation; in fact, chromosomal DNA has been identified in exosomes from cell culture supernatants, as well as in human and mouse biological fluids^37–39^. These discoveries have driven specific interests in exosome DNA for use as liquid biopsies, to facilitate the diagnosis and prognosis of cancer patients^39^. In the same way, DNA fragments present in BF could be used to investigate Mendelian monogenetic diseases in embryos, by a minimal invasive procedure^40^. In spite of this, embryo quality and implantation potential represent complex phenotypes, difficult to assess with DNA analysis. MiRNAs in BF could open up an additional possibility to investigate embryo quality in IVF cycles, as demonstrated by studies on extracellular miRNA profiles on different complex pathologies such as cancer, neurodegenerative and cardiovascular diseases^41^. Of course, further studies will be necessary to demonstrate the power of BF miRNAs to evaluate embryo quality.

## Methods

### Blastocoel Fluid Collection

As in the standard clinical practice, blastocentesis was performed before the blastocyst cryopreservation in order to prevent ice crystal formation, therefore BF used in our experiments represents discarded material^1^. BF samples from human embryos on the fifth day of development were obtained from patients undergoing to IVF cycles at the IVF Unit, Cervesi Hospital Cattolica, (Rimini, Italy) and the IVF Unit, PTA “F Jaia”, Conversano (Bari, Italy). Informed consent was obtained from the couples, and the experiments were performed in accordance to the principles set out in the World Medical Association Declaration of Helsinki. The study has been approved by the Ethics Committee of the Area Vasta Romagna. The aspiration of BF was carried out by a micropuncture through the mural trophectoderm until the blastocyst was fully collapsed around the pipette^42^. For morphological and molecular analysis, around 0.3–0.5 nL of BF isolated from each blastocyst were transferred to a PCR tube with 5 μl of RNase-free water and stored at −80 °C until further processing. In the Fig. 1, we summarized the techniques and the number of samples used (Fig. 1).

### MicroRNAProfiling of BF Using TaqMan Low-Density Arrays

#### miRNA Isolation, Reverse Transcription, Preamplification and Real-Time PCR

Nine BF samples, sent to the Catania laboratory,were analyzed for the expression of 384 miRNAs by TaqMan Low-Density Array (TLDAs) technology (Panel A) (Applied Biosystem). Because of the low quantity of samples, no procedure of microvesicles purification has been performed (Fig. 1). This highly specific technology amplifies only mature miRNAs^43^. According to a previously published protocol, samples were incubated for 1 min at 100 °C to release nucleic acids^44^. Every sample was directly reverse transcribed, without prior RNA purification, using TaqMan MicroRNA Reverse Transcription Kit and Megaplex RT Primers, Human Pool A (Applied Biosystems) in a final volume of 7.5 µl. Preamplification of cDNA from the RT reaction product, using MegaplexPreAmp Primers Pool A and TaqManPreAmp Master Mix (2x; Applied Biosystems), was run in a final volume of 25 µl. Preamplified products were loaded onto TLDAs, TaqMan Human MicroRNA Array A v2.0 (Applied Biosystems). Quantitative RT-PCR reactions were performed on a 7900HT Fast Real Time PCR System (Applied Biosystems) as follows: 94.5 °C for 10 min, followed by 40 amplification cycles of 97 °C for 30 sec and 59.7 °C for 1 min (Fig. 1).

### Expression Data Analysis

miRNA expression profiles were analyzed after a preliminary inspection of the amplification plots, using real-time RQ Manager software v1.2 (Applied Biosystems). Only miRNAs having Ct values below 35 and detected in at least 33% of biological replicates were considered expressed. To normalize miRNA profiling data, median and average expression of the plate and the pairwise Pearson correlation (r) for all miRNAs were calculated to identify the candidate stable miRNAs that showed constant expression levels among individual samples. The Ct values were input directly into the online tool RefFinder (http://leonxie.esy.es/RefFinder/?type=reference) which integrates 4 different computational algorithms (BestKeeper, comparative ΔCt method, NormFinder and GeNorm), to compare the stability of 4 candidate miRNAs and select the endogenous controls. Based on the rankings from each method, RefFinder assigns an appropriate weight to an individual gene and calculates the geometric mean of these weights to generate a final overall ranking^45^. Stability analysis revealed miR-372 (1.32) as the most stable normalization candidate in the data set, having the lowest stability values. The lower the values, the more stably expressed are the reference genes. MiR-372 was followed by miR-374 (1.86), miR-371-3p (3.13), miR-34a (3.16) and miR-373 (4.16). Expression data in the Result section normalized for miR-372 are shown as (−) ΔCt values (Fig. 2A and Table S1).

### Droplet Digital PCR

In order to support the TLDA relative quantification of miRNAs, a customized droplet digital PCR assay was used to amplify miR-17, miR-372 and miR-519d. Briefly, 22 μL of reaction mixture was prepared by adding 11 μL of ddPCR Supermix for probes (no dUTP) (cat. n. 1863010 – Bio-Rad), 1 μL of TaqMan primer/probe mix specific for each miRNAs (cat. n. 002308, 000560, 002403– Thermo Fisher Scientific), 5 μ of cDNA sample and 5 μl of PCR water. Twenty microliters of PCR reaction was loaded on the cartridge containing 70 μL of Droplet Generation Oil (cat. n. 1863005 - Bio-Rad Laboratories, Inc., Hercules, CA, United States) in appropriate wells, and then Droplet Generator QX200 was used to generate droplets. Subsequently, the generated droplets were transferred to a 96-well PCR plate (Eppendorf, Hamburg, Germany) and were amplified by using C 1000 Touch Thermal Cycler (Bio-Rad Laboratories, Inc., Hercules, CA, USA). PCR amplification was carried at the following cycling conditions: 10 min at 95 °C, 40 cycles of 94 °C for 30 s, 58 °C for 1 min, followed by 98 °C for 10 min (ramp rate 2 °C/s).

After the amplification, the plate was loaded on QX200 Droplet Reader (Bio-Rad Laboratories, Inc., Hercules, CA, USA) and both positive and negative droplets were read.

Finally, the absolute quantification of each miRNA was calculated automatically by using the QuantaSoft software, version 1.7.4 (QuantaSoft, Prague, Czech Republic) as previously described^46^. The quantification of the 3 miRNAs was reported as the number of copies/µL within the ddPCR mixture.

### Computational analysis of miRNA expression data

The 89 identified miRNAs were investigated by literature analysis and bioinformatic tools, in order to explore their biological functions.

### Embryo MiRNA conserved region identification

The sequences of mature embryo miRNAs were retrieved from MirBase (http://mirbase.org/). Multiple sequence alignment was carried out using the Clustal Omega program (https://www.ebi.ac.uk/Tools/msa/clustalo/) to identify the Consensus among miRNA sequences. Finally, CLC Sequence Viewer v 6.0 (http://www.clcbio.com) was used for the analysis of the conserved motif among the different consensus strings of clustered miRNAs.

### miRNA Function Enrichment Analysis

We compared BF miRNAs with miRNAs expressed in human MII oocytes^23^ and in spermatozoa, retrieving the last data from SpermBase (http://spermbase.org/). Moreover, BF miRNAs were checked against miRNAs annotated on the web-based resource ExoCarta (http://www.exocarta.org/). Gene Ontology (GO) and Kyoto Encyclopedia of Genes and Genomes (KEGG) pathway analysis of BF miRNAs were carried out with the Diana-miRPath v3.0 (http://snf-515788.vm.okeanos.grnet.gr/) selecting for validated targets retrieved from Tarbase. BF miRNAs were analyzed for GO enrichment in terms of the Biological Process categories applying a P-value cut-off of 0.05. The FDR method was implemented to select the biological pathways with a threshold of significance defined by P < 0.05 and a microT threshold of 0.8.

### Exosome Identification and Quantification

#### Scanning Electron Microscopy

Three BF samples diluted in 5 µl of RNase-free water were fixed in 50 µl of 3% formaldehyde-0.1% glutaraldehyde in 0.1 M phosphate buffer overnight at 4 °C. A drop of suspension (5 µl) was layered on a sterile cover glass coated with 0.1% poly-L-Lysine, postfixed in 1% osmium tetroxide (Merck, Darmstadt, Germany) in the same buffer for 1 hr at 4 °C and washed in phosphate buffer. After dehydrating in graded ethanol and critical point drying, the samples were sputtered with a 5 nm gold layer using an Emscope SM 300 (Emscope Laboratories, Ashford, UK) and then observed. This metal coating procedure avoids damaging the sample and the occurrence of charging effects. SEM images are acquired with a SUPRA 25 ZEISS microscope at a working distance of 3–5 mm, with an accelerating voltage of about 3 kV by using an in-lens detector, attesting the presence of small vesicles superimposed onto the Au morphology. The diameter distribution of BF vesicles was measured from the SEM microscopies by software processing using the Feret’s diameter function of ImageJ (https://imagej.nih.gov/ij/index.html). This parameter measures for each particle the longest distance between two points along the particle boundary. Fitting the frequency count statistic, an average BF vesicles diameter of 75 ± 3 nm and a full width at half maximum (FWHM) of 38 ± 8 nm were calculated. These values are in quite good agreement with our supplementary analyses by Nanoparticle Tracking Analysis (NTA) and with the standard reported in literature^47^.

### Nanoparticle tracking analysis (NTA)

Measurements of particle size distribution and concentration on BF samples and exosomes purified from human FF (positive control) were performed with a Nanosight NS300 system (Malvern Instruments Company, Nanosight, and Malvern, UK) based on a Nanoparticle Tracking Analysis (NTA). Briefly, BFs from 3 blastocysts were pooled, homogenized by vortexing and analyzed by a NanoSight NS300. For the analysis, BF samples were diluted 1:70 and FF Exosomes 1:100 in sterile phosphate saline buffer (PBS) to reach the optimal volume for NTA. Measurements were performed at room temperature ranging from 24.6–24.9 °C, with a Blue 488 nm laser and a sCMOS camera in several repeats. Sample analysis was conducted for 10 minutes under the following camera settings and processing conditions: Shutter 1300, Gain 512, camera level 16, NTA 3.2 Dev Build 3.2.16 and Detection Threshold 4.

### Transmission Electron Microscopy

Three BF samples diluted in 5 µl of RNase-free water were fixed in 50 µl of 3% formaldehyde–0.1% glutaraldehyde, layered on formvar copper-coated nickel grids (Electron Microscopy Sciences, Fort Washington, PA) and allow to dry for 20 min to absorb exosomes. The grids, washed in PBS, were negatively stained with 4% uranyl acetate for 5 min. For immunoelectron microscopy labelling, the grids with absorbed exosomes side down, were rinsed for 2 × 2 min with PBS and transferred in a TBS (Tris buffered saline pH 7.4) solution containing 1% BSA (bovine serum albumin) (TBS/BSA) for 10 min. at room temperature. Then the grids were incubated in blocking solution 5% BSA for 1.30 hr at room temperature, rinsed with PBS and incubated in a humid chamber overnight at 4 °C with a mouse monoclonal antibody CD81 (Santa Cruz Biotechnology, Heidelberg, Germany) in a dilution 1:50 with TBS/BSA. After washing for 3 × 3 min with TBS/BSA, the grids were stained with a 10 nm gold-labelled secondary antibody antimouse IgG (Sigma- Aldrich, S.r.l., Milan, Italy) in a dilution 1:5 with TBS/BSA at 37 °C for 1 hr in the dark. The grids were rinsed 2 × 2 with TBS/BSA, 2 × 2 with water and fixed with 1.5% glutaraldehyde in PBS for 10 min. at room temperature. After rinsed again with water, the grids were stained with 4% uranyl acetate for 5 min. and allowed to air-drying. Negative controls were prepared in the absence of primary antibody but with secondary antibody conjugate. Observations were carried out using a Jeol JEM2010 transmission electron microscopes operating at 200 kV.

### ELISA Exosome Protein Quantification

BF vesicles were analyzed for the presence of CD63 exosomal marker with a commercially available Elisa kit, ExoTEST^TM^ Ready to Use Kit (Hansa Bio Med Life Sciences Ltd). ExoTEST^TM^ Ready to Use Kit is a double sandwich Elisa assay and sensitive method for quantitative and qualitative analysis of exosomes from a small amount of human biological fluids. This consists of Elisa plates pre-coated with proprietary pan-exosome antibodies enabling specific capture of exosomes from different biological samples (http://www.exotest.eu/online_orders/proteomic-kits/elisa-rtk). Quantification of exosomal proteins was subsequently performed using appropriate detection antibodies against exosome-associated antigens (in this case tetraspanin CD63). Lyophilized Exosome standards, characterized for protein content and particle number allow the quantification of an unknown sample by a standard calibration curve. BF from 12 human blastocysts were pooled, diluted to 200 μl in PBS, divided into two aliquots and processed according to the manufacturer’s guidelines. The Protein amount was determined by reading the optical density on a Synergy™ 2 Microplate Reader (BioTek Instruments, Inc) at 450 and 570 nm. Exosomal protein amount was plotted against the standard curve created with the kit. Exosome pellet (100 μl) purified from three human FF samples by ultracentrifugation was used as a positive control. The results are expressed as the mean ± SEM for each concentration (µg/ml).

## Electronic supplementary material

Table 1S

Table 2S
